# Top Cited 2018–2019 Papers in the Section “Polymer Theory and Simulation”

**DOI:** 10.3390/polym13010043

**Published:** 2020-12-24

**Authors:** Martin Kröger

**Affiliations:** Polymer Physics, Department of Materials, ETH Zurich, Leopold-Ruzicka-Weg 4, CH-8093 Zurich, Switzerland; mk@mat.ethz.ch

This editorial deals with the most cited papers published in the years 2018–2019 in the section “Polymer Theory and Simulation” of the journal *Polymers*. They are mainly regular research articles, but two reviews are also present in this analysis. The main topics appear related to (nano)composites, entangled polymers, ring polymers, nanotubes, polyelectrolytes, brushes and polymers at interfaces. The most used simulation methods are molecular dynamics, Brownian dynamics, and Monte Carlo, with the only exception being a paper dealing with metamaterials. The evolution of the fraction of publications falling into one of these fields over the past 50 years is visualized in Figure 1. Figure 2 shows the same data differently, so that the focus is on the qualitative rather than quantitative evolution within areas of research. In the following, and inline with the spirit of this short editorial we are going to review the most cited works in the order of their number of citations, rather than by discipline.

In the most cited contribution, Zhang et al. [1] studied the mechanical properties of rice husk biochar reinforced high density polyethylene composites. Rice husk biochar has the potential to sequester carbon into more stable soil organic carbon. It was utilized in this work to reinforce high-density polyethylene and to prepare their composites by an extrusion method. Morphologies, non-isothermal crystallization behavior, and mechanical properties of the composites were successfully investigated.

Issa and Luyt [2] reviewed the kinetics of alkoxysilanes and organoalkoxysilanes polymerization. Their literature review attempts to be a comprehensive and more technical article in which the kinetics of alkoxysilanes polymerization are discussed. The kinetics of polymerization are controlled by primary factors, such as catalysts, water/silane ratio, pH, and organo-functional groups, while secondary factors, such as temperature, solvent, ionic strength, leaving group, and silane concentration, also have an influence on the reaction rates. They also summarized the study of reaction mechanisms by using ab initio and density functional theory methods alone, and in combination with molecular dynamics or Monte Carlo methods.

Fei et al. [3] investigated the morphological structure, rheological behavior, mechanical properties and sound insulation performance of thermoplastic rubber composites reinforced by different inorganic fillers. Two microscale particles, CaCO${}_{3}$ and hollow glass microspheres, were chosen to not only enhance the sound insulation but also reinforced the mechanical properties. The improved sound insulation performances of the composites have been attributed to the enhanced reflection and dissipate sound energy in the heterogeneous composite.

Sefiddashti et al. [4] studied the individual, transient and steady-state molecular dynamics of an entangled polyethylene melt undergoing steady shear flow. Under steady shear conditions, four regimes of flow behavior were evident and characterized in detail. The comparison of transient shear viscosity, with the dynamic responses of key variables of the tube model, including the tube segmental orientation and tube stretch revealed that the stress overshoot and undershoot in steady shear flow of entangled liquids are essentially originated and dynamically controlled by the shear component of the tube orientation tensor, rather than the tube stretch, over a wide range of flow strengths.

Karatrantos et al. [5] reviewed the current modeling attempts of entangled polymer diffusion in melts and nanocomposites. The focus in on modeling studies of the fundamental problem of entangled (reptational) homopolymer diffusion in melts and nanocomposite materials in comparison to experiments. Models with soft potentials or slip-springs rendered them able to estimate tube model predictions in polymer melts, enabling us to reach larger length scales and simulate well entangled polymers.

Singh et al. [6] optimized the mechanical properties for polyoxymethylene/glass fiber/polytetrafluoroethylene composites using a response surface methodology. To this end, they investigate the effects of micro-particles on mechanical properties of polyoxymethylene composites. Good correlation between experimental and response surface methodology models was obtained using normal probability plots.

Zhang et al. [7] studied the rheological behavior of amino-functionalized multi-walled carbon nanotube/polyacrylonitrile concentrated solutions and the crystal structure of composite fibers. Differential scanning calorimetry analysis demonstrated that the cyclization reaction in composite fibers have broad exothermic temperature range and low exothermic rate. These results indicate that the addition of amino-carbon nanotubes into polyacrylonitrile precursor fibers is beneficial to control the process of thermal stabilization and obtain the higher performance of composite fibers.

Rathee et al. [8] focused on explicit ion effects on the charge and conformation of weak polyelectrolytes. A comprehensive picture of polyelectrolyte titration under relevant conditions is currently lacking, due to the complexity of systems involved in the process. This work furthers knowledge in this limit utilizing hybrid Monte Carlo-molecular dynamics simulations to investigate the influence of salt concentration, pKa, pH, and counterion valence in determining the coil-to-globule transition of poorly solvated weak polyelectrolytes. These simulations serve as an essential starting point in understanding the complexation between weak polyelectrolytes and ion rejection of self-assembled copolymer membranes.

Wang et al. [9] studied the influence of temperature on the mechanical properties and reactive behavior of aluminum-polytetrafluoroethylene under quasi-static compression. Their results show that both the mechanical property and reactive behavior of these systems are strongly temperature-dependent. The material undergoes a brittle-ductile transition associated with a temperature-induced crystalline phase transformation of the matrix. They were able to characterize fracture mechanisms.

Pan et al. [10] investigated the mechanical properties of silicone/phosphor composite used in light emitting diodes package, using tensile and compression tests, via experiment and simulation. The experimental results of the tensile and compression test show that the Young’s modulus increases with the mass fraction of phosphor in silicone. Longer aging time stiffens the silicone composite and weakens the ductility of the materials. A three-dimensional model used cohesive zone material between the interface of the phosphor particles, and matrix silicone is built up to study the degradation mechanism at a micro-scale level. The simulation results indicate that the diameter of particles in silicone also impacts its interface debonding and crack growth.

Hou [11] determined the entanglement mesh size through recording the monomer mean-square displacement in entangled model polymer melts. The tube step length is measured from the intersection of the slope-1/2 line and the slope-1/4 line in a log-log plot, and the tube diameter is obtained from the data as well.

Zheng et al. [12] studied the curing kinetics and tensile properties of silica-filled phenolic amine/epoxy resin nanocomposites, using differential scanning calorimetry. Their contribution on the effects of nano-SiO${}_{2}$ particles on the tensile properties and tensile fracture face morphology of nanocomposites show that the uniform dispersion of these nanoparticles plays an important role in promoting the tensile performance of nanocomposites.

Chremos and Douglas [13] characterized the influence of branching on the configurational, packing, and density correlation function properties of entangled polymer melts of linear and star polymers, with emphasis on molecular masses larger than the entanglement molecular mass of linear chains. They introduced a model of entanglement phenomenon in such polymers that assumes polymers can viewed in a coarse-grained sense as soft particles and, correspondingly, they model the emergence of heterogeneous dynamics in polymeric glass-forming liquids to occur in a fashion similar to glass-forming liquids, in which the molecules have soft repulsive interactions. Based on this novel perspective of polymer melt dynamics, they propose a functional form that can describe their simulation results.

Lee and Jung [14] conducted a Monte Carlo study of a dynamically constrained lattice model to investigate the slow dynamics of ring polymer melts, created by an asymmetric interaction of threading configurations. In their work they constructed a lattice model under the assumption of asymmetric diffusivity between two threading rings, and investigated a link between the structural correlation and its dynamic behavior. They observe abnormally slow dynamics and found that the decoupling between internal structure relaxation and diffusion is crucial to understand the threading effects. As the the length of chains increases, the ring diffusion abruptly slows down to the glassy behavior, which is supported by a breakdown of the Stokes–Einstein relation.

Shchetnikava et al. [15] performed a comparative analysis of five different tube models for the linear rheology of monodisperse linear entangled polymers. To this end, they gathered a large set of experimental data. The comparison allowed them to highlight and discuss important questions related to the relaxation of entangled polymers, such as the importance of the contour-length fluctuations process and how it affects the reptation mechanism, or the contribution of the constraint release process on the motion of the chains. Based on these comparisons, they propose a modification to the time marching algorithm.

Jehser et al. [16] investigated the scaling and interactions of linear and ring model polymer brushes via dissipative particle dynamics simulations. To this end they varied the system parameters such as chain length, surface coverage, solvent conditions, and the strength of a bond repulsion potential that helps to prevent excessive overlap.

Zhou et al. [17] focused on the entropy-induced separation of binary semiflexible ring polymer mixtures in spherical confinement. Coarse-grained molecular dynamics simulations are used to investigate the conformations rings of two different lengths confined in a hard sphere. With a low number density or a weak bending energy of the long chains, long chains are immersed randomly in the matrix of short ones. As the number density and bending energy of the chains is increased up to a certain threshold, a nearly complete segregation between long and short chains is observed. The explicit segregated structures of the two components in spherical confinement are induced by a delicate competition between the entropic excluded volume (depletion) effects and bending contributions.

Tsamopoulos et al. [18] studied the steady state shear rheology of unentangled and marginally entangled ring polymer melts from large-scale nonequilibrium molecular dynamics atomistic simulations. For all melts studied, rings are found to exhibit shear thinning but to a lesser degree compared to linear counterparts, mostly due to their reduced deformability and stronger resistance to alignment in the direction of flow. They demonstrated that long time dynamics are strongly heterogeneous both for rings and (especially) linears, but that the applied flow field significantly suppresses dynamic heterogeneity. To understand these behaviors, authors computed survival times and penetration lengths, and found that the overwhelming majority of threadings under shear are extremely weak constraints, as they are characterized by very small penetration lengths, thus also by short survival times. They are expected therefore to play only a minor (if any) role on chain dynamics.

Last but not least, Zhang et al. [19] focused on the in-plane mechanical behavior of a star-re-entrant hierarchical polymer-based metamaterial, that is characterized by a tunable negative Poisson’s ratio. The network-like material has a star-re-entrant hierarchical structure, and has been investigated both experimentally and via finite elements in this work. Authors systematically explore the effects of the design parameters on mechanical behavior, indicating that high specific stiffness and large auxetic deformation can be remarkably enhanced and manipulated through combining parameters of both subordinate cell and parent re-entrant hierarchical unit cells.

## Figures and Tables

**Figure 1:**
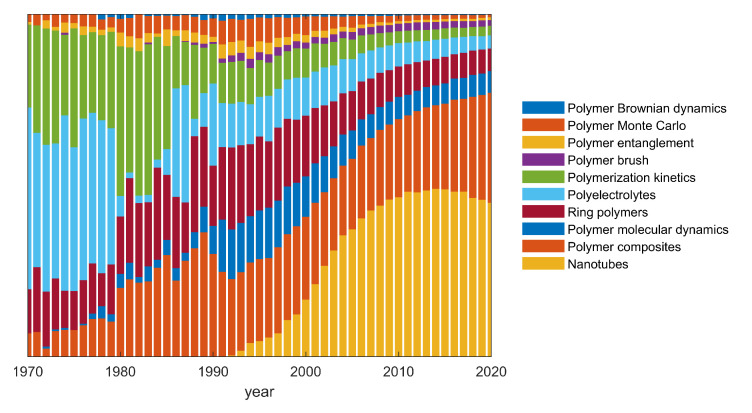
Time evolution of the fraction of publications belonging to one of the 10 listed areas of research. Data extracted from the Web of Science.

**Figure 2:**
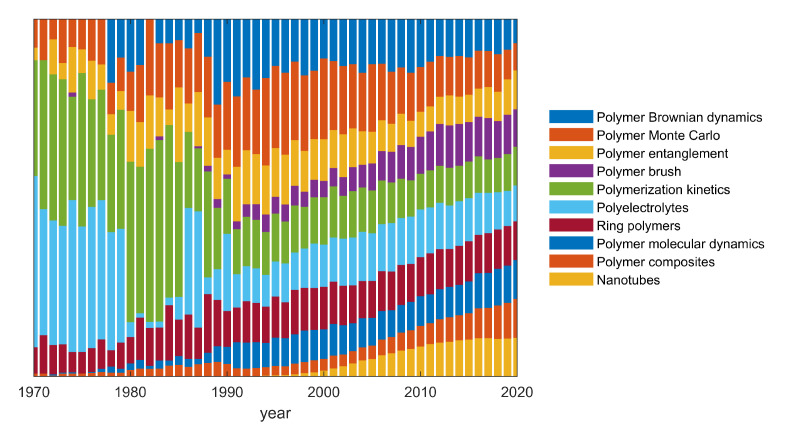
Different representation of the data shown in Figure 1. Time evolution of the fraction of publications in the 10 listed areas of research, relative to the maximum annual number of publications, which is different for each area. For all areas, the maximum was reached in the recent past; this is why the fractions became very similar in 2020, in this representation.
